# Science–graphic art partnerships to increase research impact

**DOI:** 10.1038/s42003-019-0516-1

**Published:** 2019-08-06

**Authors:** Colin K. Khoury, Yael Kisel, Michael Kantar, Ellie Barber, Vincent Ricciardi, Carni Klirs, Leah Kucera, Zia Mehrabi, Nathanael Johnson, Simone Klabin, Álvaro Valiño, Kelsey Nowakowski, Ignasi Bartomeus, Navin Ramankutty, Allison Miller, Meagan Schipanski, Michael A. Gore, Ari Novy

**Affiliations:** 1 International Center for Tropical Agriculture (CIAT), Km 17, Recta Cali-Palmira, Apartado Aéreo 6713, 763537 Cali, Colombia; 2 Independent Artist, San Jose, CA USA; 3 Department of Tropical Plant and Soil Science, University of Hawaii at Manoa, 3190 Malie Way, Honolulu, HI 96822 USA; 4 Aspen Global Change Institute, 104 Midland Ave #205, Basalt, CO 81621 USA; 5 The Institute for Resources, Environment, and Sustainability, University of British Columbia, 429-2202 Main Mall, Vancouver, BC V6T 1Z4 Canada; 6 School of Public Policy and Global Affairs, University of British Columbia, 251-1855 West Mall, Vancouver, BC V6T 1Z2 Canada; 7 World Resources Institute, 10G Street, NE Suite 800, Washington, D.C. 20002 USA; 8 SERVIR Science Coordination Office, NASA Marshall Space Flight Center, National Space Science & Technology Center, 320 Sparkman Drive, Huntsville, AL 35805 USA; 9 Grist, 1201 Western Ave., Suite 410, Seattle, WA 98101 USA; 10 Independent Author, New York, NY USA; 11 Independent Information Designer, A Coruña, Spain; 12 Independent Information Designer, San Juan, Puerto Rico USA; 13 Integrative Ecology Department, Estación Biológica de Doñana (EBD-CSIC), Avenida Américo Vespucio 26, Isla de la Cartuja, Sevilla, E-41092 Spain; 14 St. Louis University, Department of Biology, 3507 Laclede Avenue, St. Louis, MO 63103 USA; 15 Danforth Plant Science Center, 975 North Warson Road, St. Louis, MO 63132 USA; 16 Department of Soil and Crop Sciences, Colorado State University, Fort Collins, CO 80523-1170 USA; 17 Plant Breeding and Genetics Section, School of Integrative Plant Science, Cornell University, Ithaca, NY 14853 USA; 18 San Diego Botanic Garden, 230 Quail Gardens Drive, Encinitas, CA 92024 USA

**Keywords:** Agriculture, Policy, Education

## Abstract

Graphics are becoming increasingly important for scientists to effectively communicate their findings to broad audiences, but most researchers lack expertise in visual media. We suggest collaboration between scientists and graphic designers as a way forward and discuss the results of a pilot project to test this type of collaboration.

When we think of groundbreaking scientific advances, it is often in visual terms – the first depictions of the structure of DNA; Darwin’s sketches of the tree of life; even DaVinci’s *Vetruvian Man*. The power of these pictures to speak to people, especially those outside our specialized research communities, is worth far more than a thousand words.

Scientists’ need for visual art has never been greater. More sophisticated graphics are required to communicate the results of ever more complex and transdisciplinary research. Well-constructed graphics can widen the impact of research articles striving to be noticed in an ever-increasing flood of published work, and supplementary visuals, for instance graphical abstracts, are often now requested by journals, if not required^1^. Funders are also increasingly emphasizing the value of graphics in grant proposals^2^. Online, where viewers decide whether to engage with material within a matter of seconds^3^, compelling visuals are pivotal, especially as research organizations incorporate social media attention in their impact metrics.

While many researchers are rising to the challenge of communicating their work via social media and other formats beyond their traditional channels^4^, very few scientists have expertise in visual media communications, and even fewer in design tailored for online platforms. Learning the specialized skills needed to create graphics for the changing array of conventional and new science media is a very big ask.

But scientists do not need to go it alone. Collaborations between researchers, graphic designers, and other visual communications professionals offer great potential (Box 1).

## Test project overview

Recently, we tested the efficacy of scientist–graphic artist collaborations by pairing six research laboratories involved in different aspects of biological and agricultural sciences with graphic designers and media content creators. The work of the eight participating scientists focused on complex, societally relevant subjects within biology, food, and agriculture, including pollinators and threats to biodiversity, modern plant breeding, agricultural development and land use change, phenomics and other new agricultural technologies, agricultural sustainability, and the origins and domestication processes of food plants.

The five participating artists were chosen for their track records as producers of attractive and interesting visual online media, either as graphic design professionals or as talented hobbyists. Some had research backgrounds while others had no science training. All of the scientists and graphic designers approached were enthusiastic about experimenting with this cross-disciplinary collaboration. The researchers and designers were paired based on the artists’ interests among the scientific topics, and the designers were compensated for their contributions. The scientist–artist pairs were asked to create infographics – in this case defined as visually arresting, quickly understandable, graphical representations of scientific research – based on the research laboratories’ current projects, within three months.

At the end of this time, the researchers and artists, supplemented by additional professionals and experts in graphic design and infographics, presented the collaborations and their resulting products to scientists, research organizations, and funders via an interactive communications seminar^5^ at the “Science Transcending Boundaries” AAAS annual meeting in Washington D.C. in February 2019.

## Iterative approach to collaboration

**Fig. 1:**
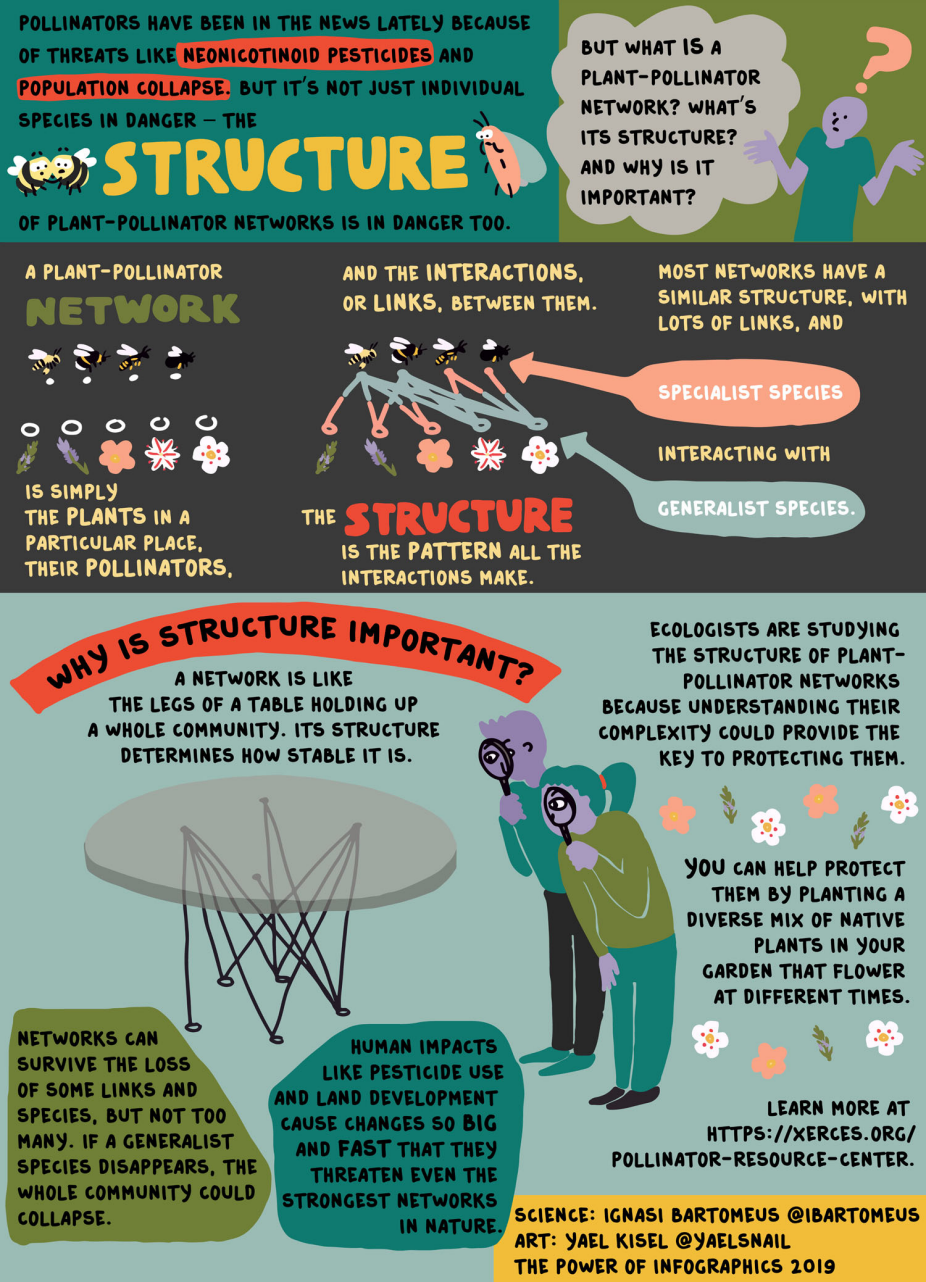
An explanation of why it’s important to protect the structure of plant-pollinator interaction networks. This graphic was designed with bright colors and a minimum of text so that it could be shared on social media. The biggest challenge was finding a way to concisely, yet clearly, explain a high-level abstract topic to biodiversity-interested but non-scientist audiences. The scientist–artist team tried many different approaches before settling on the combination of a news-related hook, a quick graphical summary, and the table metaphor. To reach intended audiences, the graphic was produced both in English and in Spanish. Design by Yael Kisel based on the research of Ignasi Bartomeus [Estación Biológica de Doñana (EBD-CSIC)]

The collaborations typically began with conversations aimed at *identifying the target audience*. This was surprisingly challenging for a number of the researchers, who wanted to communicate to “the general public”. Because the artists knew that different audiences require different approaches, they challenged the scientists to be as specific as possible. The teams eventually arrived at much more refined audience targets, e.g. “English and Spanish speaking viewers already interested in biodiversity conservation” (Fig. 1).

**Fig. 2:**
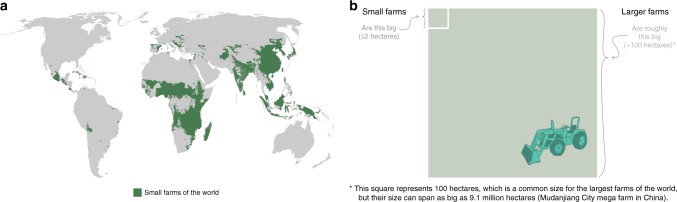
Two designs from the same infographic focused on the role of small farms in the global food system. **a** is a data visualization of specific data from the research representing the global geography of small farms. **b** is a representation of differences in farm size definitions, a concept that the artist thought was more effectively communicated through abstraction. Design by Ellie Barber based on the research of Vincent Ricciardi, Zia Mehrabi, and Navin Ramankutty (University of British Columbia). The full infographic is available in the Dryad Digital Repository

These conversations fed the next step of co-creation, *refining the messages* of the infographics. In many cases the middle ground had to be found between the scientists’ conviction that the graphics accurately and comprehensively represented the data, and the artists’ emphasis on streamlining the messages to make them easier to understand. Each team had to determine how to distill the research into a communicable story without simplifying to the point that key context was lost. For some, the compromise was found by including data visualizations, to communicate specific information, as well as more abstract designs to relay broader concepts (Fig. 2). For others, presentation materials created by the scientists themselves were adapted and further developed into visual components (Supplementary Fig. 1).

In every case, the process of refining the message and then creating the graphic was *iterative*, as the teams tried different arrangements of information in search of an effective story. Often the supporting, and even the main, messages changed as the work progressed and as the artists provided input on what they found easy to communicate and on what they thought would be relevant to the target audience. In some cases, the message refinement processes brought forward points that the scientists originally thought were too obvious to mention (Supplementary Fig. 2). Colleagues, friends, and family from both the scientists’ and artists’ worlds provided litmus tests for progress. By the end of the project, all of the teams were pleased with their products, which they thought were scientifically accurate, visually appealing, and effectively communicated. All of the infographics are available in the Dryad Digital Repository^6^.

A number of the participating researchers were surprised to find that the act of translating their work into an infographic pushed their science forward. They agonized over the challenge of distilling complex concepts into clear, focused, and accessible messages, but the process helped them to identify the central components of their work and to note areas that they had not studied sufficiently. The process also forced the researchers to reflect on, and then communicate, why they do what they do, as well as how their work impacts society.

## Recommendations

As the presentation of science moves beyond the traditional static journal article^7^, there is every reason to think that graphic art will become ever more critical. As a result of our experience, we have developed a set of recommended actions for researchers and their institutions, for graphic art professionals, and for funders, to facilitate productive scientist–artist collaborations (Box 2).

Researchers and their institutions should recognize the value of science-graphic art collaborations in improving the communication of research and the accessibility of results relevant to society. The sooner designers are consulted during the research process the better−not only to facilitate the creation of visual media, but because these collaborations improve current and potential future research. Based on the complex research topics of the scientists involved in this project and their uniform response that their work and its communication benefited from these collaborations, we believe that scientists in most, if not all, research areas would similarly benefit. Research societies and journals can support scientist-artist collaborations through promotion and training opportunities.

During the presentation of our project at the AAAS conference, members of the audience asked more than once how they could find a skilled artist to work with. Some organizations contain dedicated arts/design/communications offices that can work with researchers to develop graphics to increase impact (e.g.^8–10^). For scientists without this institutional support, the continued creation and expansion of networks (e.g.^11^), organizations, and companies (e.g.^12^) providing these services would be of tremendous value.

Finally, funders should look positively on broader impacts budgets in grant proposals that include resources for graphic design, and should explicitly name graphic design components as broader impacts work they will support. We believe that the relatively limited additional funding needed would provide substantial returns in impact.

Box 1 benefits, applications, and challenges of scientist-graphic artist collaborations **Benefits**

- Better communication of scientific findings
- Increased awareness of research by both experts and non-experts
- Greater impact and reach of science

**Applications**

- Infographics
- Conference posters
- Graphical abstracts
- Journal article figures
- Journal article covers
- Magazine and newspaper graphics
- Website, blogs, and social media graphics
- Public art pieces and murals
- Scientific, policy, outreach, and educational presentations
- Videos and animations

**Challenges**

- Additional time required for collaboration with graphic artists
- Additional project costs to support graphic artists

Box 2 recommendations for fostering scientist–graphic artist collaborations **Researchers and institutions**

Promote science-graphic art collaborations by including, engaging, and supporting graphic artists in research projects - both for improved science communications and for the research benefits gained through the iterative collaborative process

**Graphic art professionals**

Create and expand networks, non-profit organizations, and companies that specialize in producing scientific graphics and/or help researchers to identify artist collaborators

**Funders**

Provide financial support for including graphic artists in funded projects.

## Discussion

Graphics have the potential to increase the attractiveness, understandability, and communication power of research findings. They can help science reach audiences that research literature never will. As such, they are a tremendous asset in a time when the increased politicization of complex scientific issues, such as the future of food and nutrition security, necessitates the communication of science to society in ways are accessible and engaging.

Scientist-artist collaborations can certainly improve traditional research visuals, such as journal figures, presentations, and posters. But applications aimed at reaching broader audiences – online, in print, and on the street – have the potential to do much more (Box 1).

As with any multidisciplinary work, such collaborations are not without cost – both in terms of the extra time needed for the iterative process to be productive, and the additional financial resources required to fairly compensate graphic professionals for their contributions. We found that the collaborations necessitated multiple rounds of idea generation and then further concept refinement, but the investment paid off in terms of powerfully communicated graphic art and scientists’ clearer conceptualizations of their own work. In our view, the benefits of scientist-artist collaborations far outweigh their costs – especially as scientific organizations, journals and other media, and funders continue to ask more of researchers with regard to graphics, broader impacts, and public outreach.

## Supplementary information

Supplemental Material

## Data Availability

All infographics produced in this project available from the Dryad Digital Repository: 10.5061/dryad.7j5d5t0^6^.
