# Supplementary material for: Science–graphic art partnerships to increase research impact
Source: Commun Biol. 2019 Aug 6;2:295. doi: 10.1038/s42003-019-0516-1 (PMC6684576; doi:10.1038/s42003-019-0516-1)
Supplement: Supplementary file 1 — Supplemental Material [file 42003_2019_516_MOESM1_ESM.pdf]

Supplementary Materials

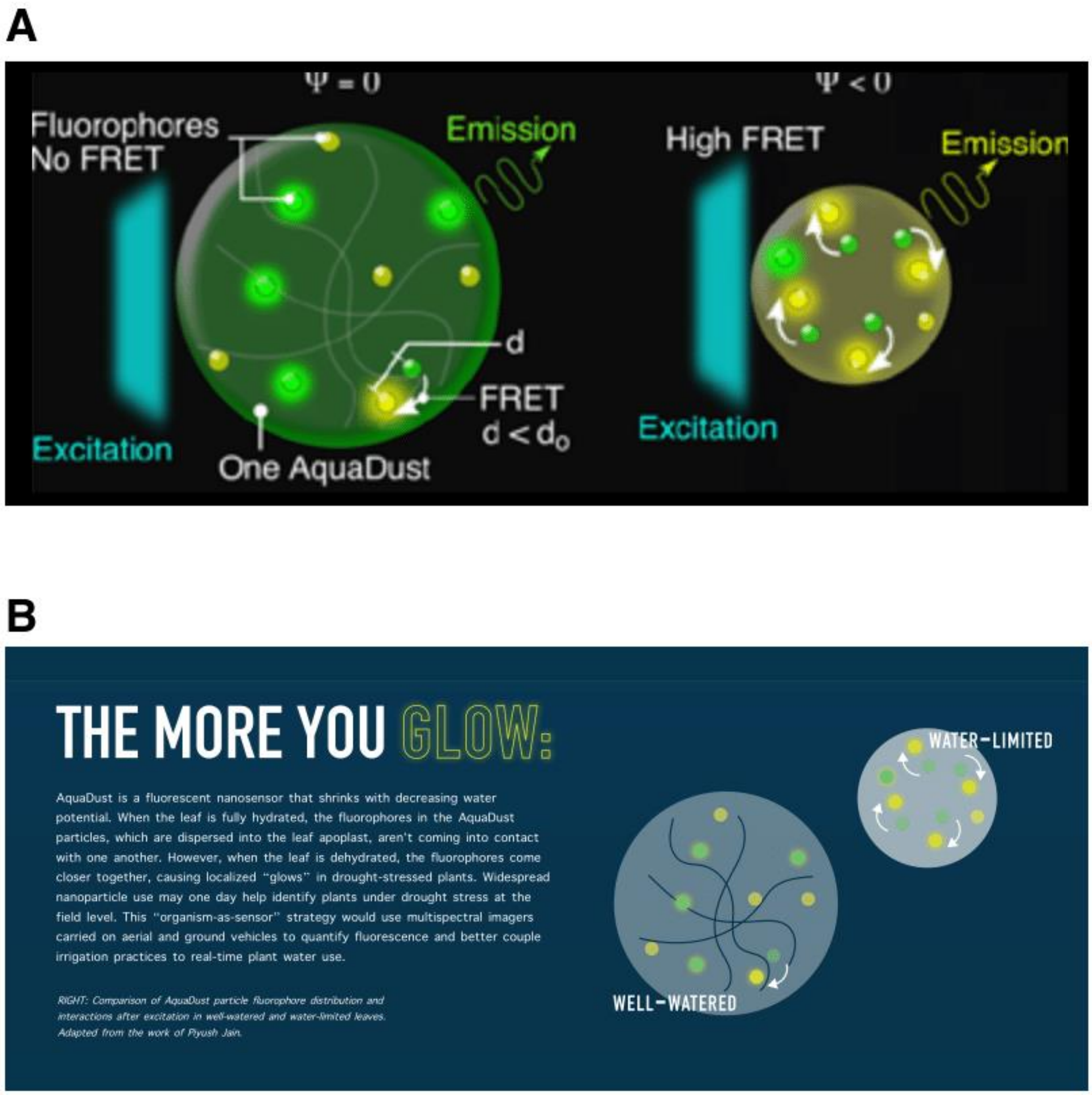

Supplementary Figure 1. An example of a visual adapted from the researcher's slide deck into an infographic focused on harnessing new technologies to improve crop resilience. After several rounds of consultations with the collaborating researcher, the artist remade the figure for the panel, simplifying the details and making the concept more understandable by a non-scientific audience while keeping key features. The choice of header text (The More You Glow) also helped to aid in conceptual understanding of the "glowing" nanoparticles providing an in planta measurement of leaf water potential under water-limited conditions. Design by Leah Kucera based on the research of Michael Gore (Cornell University).

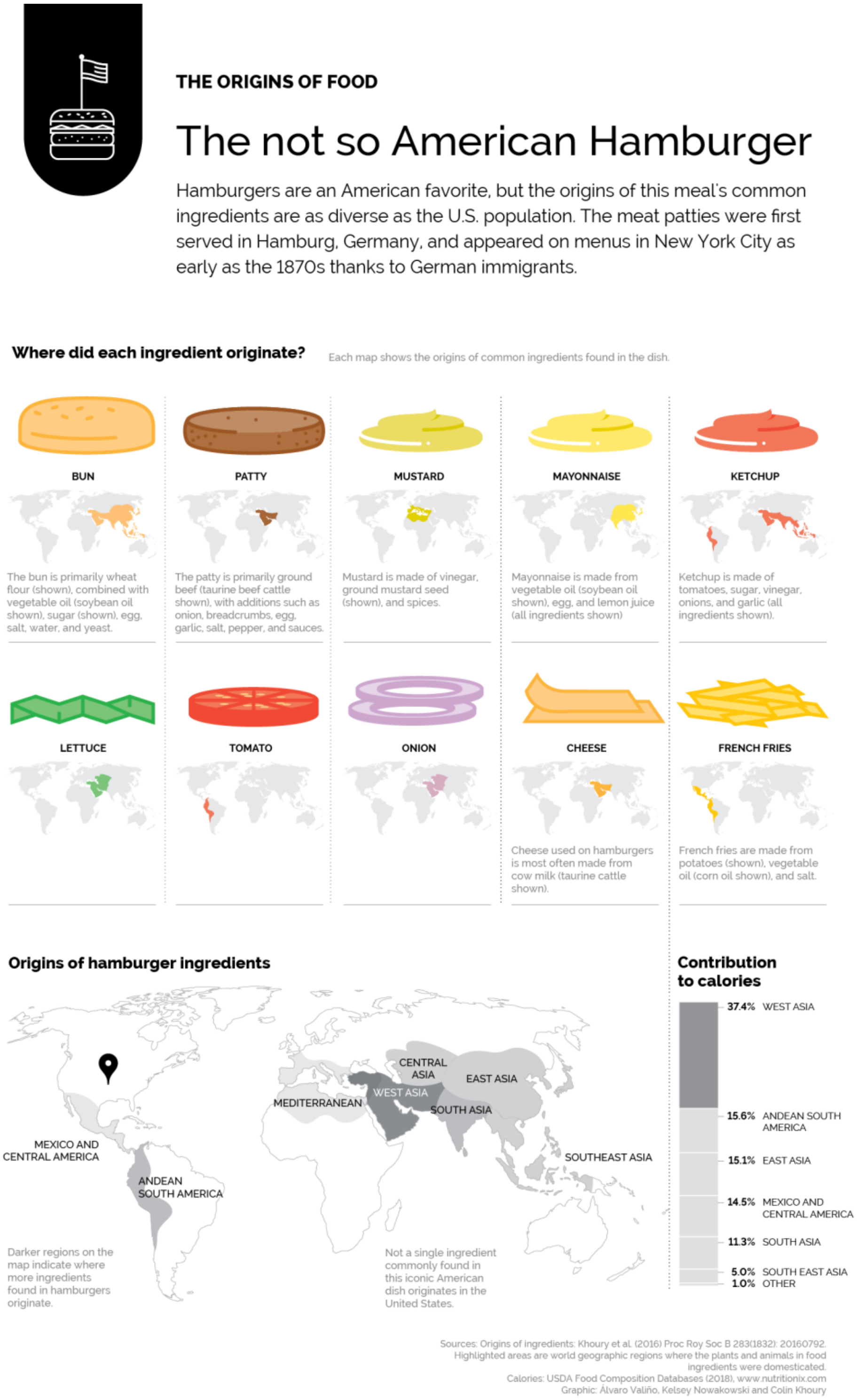

Supplementary Figure 2. An infographic breaking down the main ingredients in an internationally recognized dish associated with a particular country, showing where the plants and animals in these ingredients were first domesticated. The scientist was surprised during the co-creation process at the artists' insistence that a main map be included, with clear descriptions of world regions as well as a place marker for the country of the dish. Feedback from viewers of this infographic confirmed the central importance of the map to interpreting the message. Design by Álvaro Valiño and Kelsey Nowakowski based on the research of Colin K. Khoury (CIAT).
